# Volume-Rendering-Based Interactive 3D Measurement for Quantitative Analysis of 3D Medical Images

**DOI:** 10.1155/2013/804573

**Published:** 2013-05-16

**Authors:** Yakang Dai, Jian Zheng, Yuetao Yang, Duojie Kuai, Xiaodong Yang

**Affiliations:** Suzhou Institute of Biomedical Engineering and Technology, Chinese Academy of Sciences, No. 88 Keling Road, New District, Suzhou 215163, China

## Abstract

3D medical images are widely used to assist diagnosis and surgical planning in clinical applications, where quantitative measurement of interesting objects in the image is of great importance. Volume rendering is widely used for qualitative visualization of 3D medical images. In this paper, we introduce a volume-rendering-based interactive 3D measurement framework for quantitative analysis of 3D medical images. In the framework, 3D widgets and volume clipping are integrated with volume rendering. Specifically, 3D plane widgets are manipulated to clip the volume to expose interesting objects. 3D plane widgets, 3D line widgets, and 3D angle widgets are then manipulated to measure the areas, distances, and angles of interesting objects. The methodology of the proposed framework is described. Experimental results indicate the performance of the interactive 3D measurement framework.

## 1. Introduction

Modern medical imaging (such as CT, MRI, and optical CT) can produce 3D images, which have been widely used to assist qualitative and quantitative diagnosis in clinical applications. One of the commonest needs in these image-guided diagnoses is quantitative measurement of the interesting object in the image. Although various automated and semiautomated image processing methods (such as segmentation and registration) have been proposed to improve measurement efficiency, they have not achieved the accuracy by interactive measurement with human experience. Therefore, currently interactive measurement methods are still commonly used in practical applications. However, most existing methods perform interactive measurement on each of the three orthogonal slices. This way is not intuitive enough and requires the operator to reconstruct stereoscopic structure of the interesting object in his mind. The idea to directly perform interactive measurement in 3D space is thus inspired, where volume visualization technology should be used. 

Surface rendering and volume rendering are two main branches of volume visualization. After segmentation and reconstruction, surface rendering can display the extracted surface very fast with powerful graphics hardware. Compared with surface rendering, volume rendering is computationally expensive. However, it can visualize the volume directly without the segmentation and reconstruction operations. With the growing of the computational performance of the modern computer, the volume rendering is becoming very popular for volume visualization. Generally, volume rendering is just used for qualitative display of the volume. To achieve vivid and accurate 3D measurement based on volume rendering, two key problems should be addressed: (1) how to interactively explore the volume in 3D space and measure the interesting object; (2) how to display the volume, interesting object, and human-machine interaction tool in the 3D scene properly. We have proposed a volume-rendering-based interactive 3D measurement framework, which integrates 3D widgets and volume clipping with volume rendering, to address the above problems. 

Volume clipping is the commonest way to cut away unwanted parts of the volume and expose the interesting object. In [1], clipping geometries based on stencil buffer test were introduced to cut the volume. Taking advantages of 3D texture operations on graphics hardware, Sommer et al. [2] implemented arbitrary clip planes for volume exploration. Several clipping methods, such as depth-based clipping and volumetric clipping, were proposed in [3] to implement complex geometries for volume exploration. Besides, Khanduja and Karki [4] combined clipping with multiresolution rendering to visualize large-scale 3D datasets. Although these methods achieved good clipping results, they did not attempt to manipulate the clipping immersively in 3D space. Thus, we use 3D widgets, instead of traditional panel widgets such as pushbuttons and sliders, to assist immersive volume clipping and implement 3D measurement.

3D widget was introduced by Conner et al. [5]. It can be considered as an encapsulation of geometry and behavior that is used to adjust the controlled objects. Many publications have reported the use of 3D widgets in immersive volume clipping [6–11]. For instance, McGuffin et al. [12] applied 3D widgets to browse volumetric data. Huff et al. [10] developed three interactive 3D widgets (i.e., eraser, digger, and clipper) for volume sculpting and visualization. However, almost all reported volume-rendering-based immersive clipping and measurement methods (with 3D widgets) depended on texture-based volume rendering [13]. Immersive volume clipping and measurement strategies based on other volume rendering algorithms (such as ray casting, splatting, and shear warp) are seldom described. For texture-based volume rendering, the volume is resampled to multiple textured polygons (with depth information), which are then rendered in 3D space. Therefore, using texture-based volume rendering, the clipped volume, intersection images between the volume and clipping geometries, and 3D widgets can be displayed correctly in 3D space. Nevertheless, for the other volume rendering algorithms, a 2D projection image is calculated directly as the final rendering result; therefore, intermediate textured polygons with depth information cannot be obtained, making the realization of vivid clipping and measurement in 3D space challenging.

Compared with existing measurement methods, the features of our interactive 3D measurement framework are described as follows: (1) 3D plane widgets are designed to immersively manipulate volume clipping to expose interesting objects and also measure areas of interesting objects; (2) various volume rendering algorithms (such as ray casting, splatting, and shear warp) are supported, regardless of CPU-based or GPU-based implementations; (3) the clipped volume, intersection images between the volume and clip planes, and 3D widgets can be displayed correctly; (4) 3D line widgets and 3D angle widgets are designed to measure distances and angles of interesting objects, respectively. The methodology of our interactive 3D measurement framework is introduced in Section 2. Representative experimental results using the measurement framework are demonstrated in Section 3. The conclusion and future work are presented in Section 4.

## 2. Methods

**Figure 1:**
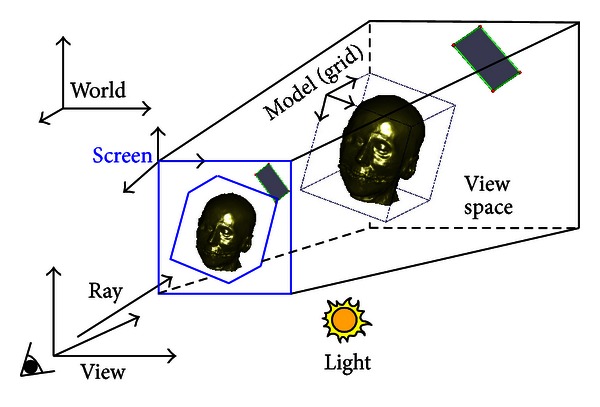
Integrated rendering environment. There are five coordinate systems. All models (such as the clipped volume, intersection images between clipping geometries and the volume, and 3D widgets) placed in the view space are rendered onto the screen.

To achieve immersive volume clipping and measurement with 3D widgets, two requirements must be fulfilled: (1) all models (such as the clipped volume, intersection images between the volume and clipping geometries, and 3D widgets) in the 3D scene should be rendered correctly; (2) the widgets should be manipulated “look right” and the parameters adjusted by the widgets should be precise. We thus place all models in an integrated rendering environment (which is illustrated in Figure 1) for accurate rendering and immersive volume clipping and measurement. It is worth noting that the center of the volume is always located in the intermediate plane (see Figure 5) of the view space.

### 2.1. Integrated Rendering Environment

There are five coordinate systems in the rendering environment, including the coordinate system of the volume grid space *G*, the coordinate system of the volume model space *M*, the world coordinate system *W*, the coordinate system of the view space *V*, and the screen coordinate system *S*. *W* is the absolute coordinate system where the volume and widgets are placed. The view space defines the visible region in *W*. The coordinate transformation from the grid space to the screen space can be written as

(1) XS=TSV·TVW·TWM·TMG·XG,

where ^*G*^ *X* is the position of each voxel in *G* and ^*S*^ *X* is the transformed coordinate value in *S*. Both of them can be denoted as [*x*,*y*,*z*,1]^*T*^. ^*M*^ *T*_*G*_, ^*W*^ *T*_*M*_, ^*V*^ *T*_*W*_, and ^*S*^ *T*_*V*_ can be written in a unified format ^*J*^ *T*_*I*_, which is a 4 × 4 matrix representing the transformation from the coordinate system *I* to the coordinate system *J*. In the environment, each widget is rendered using OpenGL directly, while the clipped volume is rendered using volume rendering (such as ray casting, splatting, and shear warp).

### 2.2. 3D Widgets

**Figure 2:**
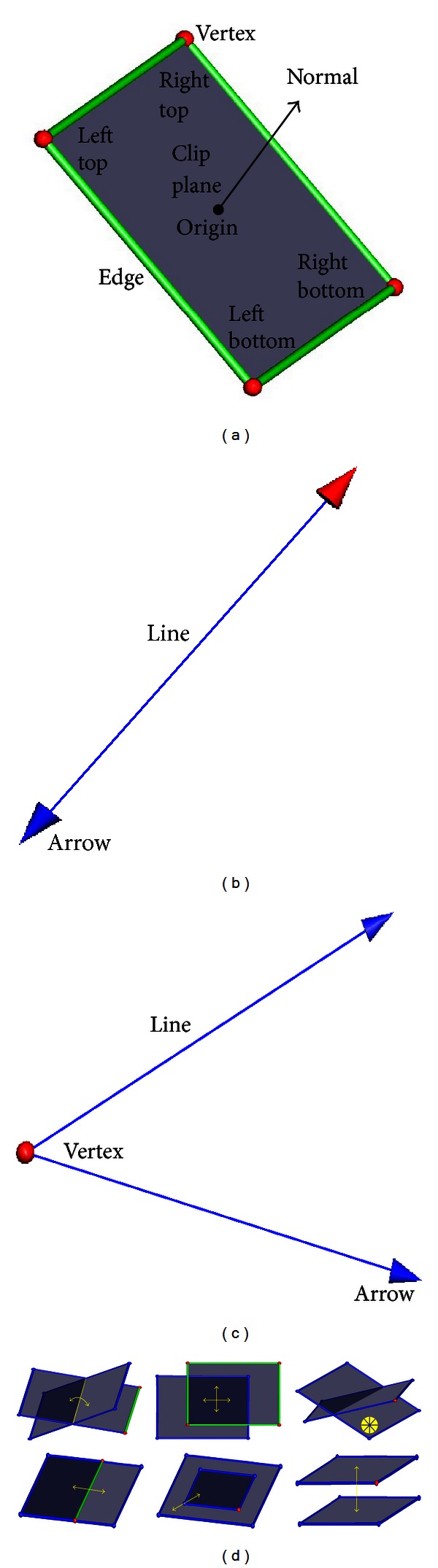
3D widgets used in our interactive 3D measurement framework. (a) 3D plane widget. (b) 3D line widget. (c) 3D angle widget. (d) Operations related to the 3D plane widget. 3D plane widgets are used to clip the volume and measure the areas of interesting objects. 3D line widgets and 3D angle widgets are used to measure distances and angles of interesting objects, respectively.

**Figure 3:**
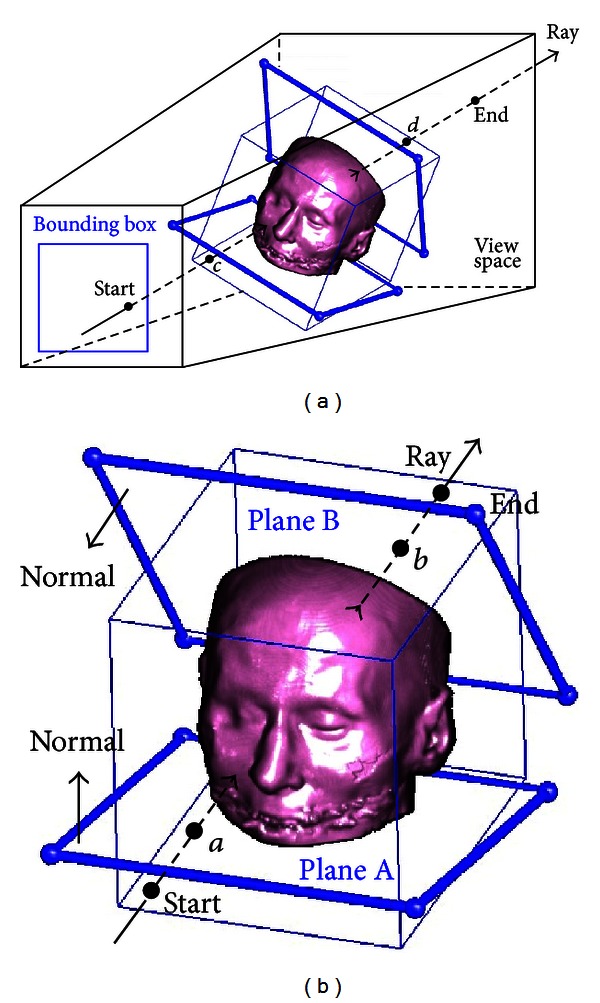
The calculation of the projection image using ray casting. (a) Intersections between the ray and volume. (b) Intersections between the ray and clip planes.

A 3D widget can be regarded as an integration of geometry and manipulation, which is used to adjust other controlled objects [12]. The 3D plane widget, 3D line widget, and 3D angle widget used in our interactive 3D measurement framework are illustrated in Figure 2. The 3D plane widget (see Figure 2(a)) is composed of a clip plane, four vertices (implemented with spheres), and four edges (implemented with cylinders). The 3D plane widget can be manipulated by 2D mouse to achieve the following operations (see Figure 2(d), from left to right, top to bottom): rotation around a central axis, horizontal translation, arbitrary rotation, extension or shrinkage, zoom, and vertical translation. Using 3D plane widgets, the volume can be clipped immersively to expose interesting objects (details on volume clipping with 3D plane widgets are described in Section 2.3). Similarly, 3D line widgets (see Figure 2(b)) and 3D angle widgets (see Figure 2(c)) can be manipulated immersively in 3D space to measure distances and angles of interesting objects, respectively.

With the selection mechanism of OpenGL, the widget in *W* can be manipulated by 2D mouse. We take the manipulation of the 3D line widget for an example to depict the implementation detail. Given that an arrow of the 3D line widget is selected and moved, the position of the arrow in the world coordinate system *W* is ^*W*^ *X*_*a*_. The previous and current positions of the mouse cursor on the screen are, respectively, [*x*_old_,*y*_old_,0,1]^*T*^ and [*x*_new_,*y*_new_,0,1]^*T*^. Then we can get the corresponding coordinates, denoted as ^*W*^ *X*_old_ and ^*W*^ *X*_new_, in *W* by

(2) XW  =  TVW·TSV·XS,

whereafter, the vector of the mouse movement in *W* can be calculated by

(3) VW=XnewW−XoldW.

Then the new position of the arrow in *W* can be obtained as follows:

(4) XaW⟵  XaW  +  VW.

The manipulations of the 3D angle widget and 3D plane widget are similar. 

Provided that the positions of the two arrows of the 3D line widget in the world coordinate system *W* are ^*W*^ *X*_*a*_ and ^*W*^ *X*_*b*_, respectively, then the distance of the 3D line can be calculated easily as ||^*W*^ *X*_*a*_ − ^*W*^ *X*_*b*_||. Given the three vertices for the 3D angle widget and four vertices for the 3D plane widget (see Figure 2), the respective angle and area can also be calculated very easily.

### 2.3. Volume Clipping with 3D Plane Widgets

The clipped volume and 3D plane widgets are rendered by two steps. First, the projection image of the clipped volume is calculated by volume rendering. Then, the projection image and 3D plane widgets are rendered to obtain the final rendering image. 

#### 2.3.1. Projection of the Clipped Volume Using Volume Rendering

We have implemented three volume renderers to obtain the projection image of the clipped volume, including *ray casting*, *splatting*, and *shear warp*, which are based on the ray-casting, splatting, and shear-warp algorithms, respectively. All of these algorithms finally can come down to the recursive front-to-back composition [7]:

(5) $$\begin{array}{c} c⟵C_{S}\cdot \alpha_{S}\cdot \left(1-\alpha \right)+c,\\ \alpha ⟵\alpha_{S}\cdot \left(1-\alpha \right)+\alpha , \end{array}$$

where *c* and *α* are, respectively, the accumulated color and accumulated opacity and *C*_*S*_ and *α*_*S*_ are, respectively, the current sampling color and sampling opacity. However, the ray-casting, splatting, and shear warp are implemented differently (i.e., image order, object order, and hybrid, resp.). The implementation details of the renderers are described as below.

*The Ray-Casting Renderer*

Figure 3 illustrates the approach used by *ray casting* to obtain the projection image. The bounding box formed by projecting the volume to the screen is figured out in advance. The accumulated color and accumulated opacity of each pixel in the bounding box are initialized to zeros. The finally accumulated value of each pixel in the bounding box is calculated as follows.

1. Cast a ray from the origin of *V* to the pixel and initialize *S* and *E* to the start and end positions of the ray in the view space, respectively.
2. Assume the ray intersects against the volume sequentially at *c* and *d* and let *S* = *c* and *E* = *d*.
3. For each clip plane, the half space of the normal is preserved, and the rest is removed.
4. Assume the two intersections between the ray and the planes are *a* and *b*, respectively. Discard the line segments out of the clip planes, and let *S* = *a* and *E* = *b*.
5. Traverse from *S* to *E*, calculate opacity and color at each sampling place, and compute the final accumulated color and accumulated opacity recursively according to (5).

After all pixels within the bounding box are processed, the projection image is obtained.

*The Splatting and Shear Warp Renderers*

The two renderers calculate the projection image based on the sheet which is an axis-aligned volume slice that is most parallel to the screen.

**Figure 4:**
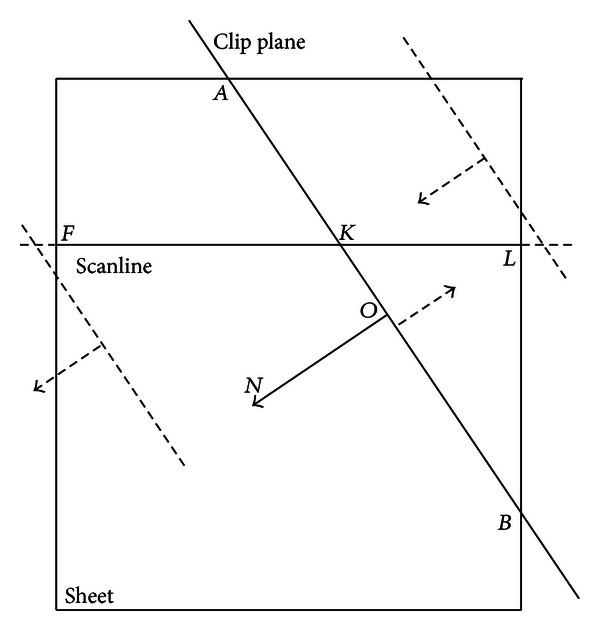
The clipping of a scanline. The segment ($\overset{\to }{FK}$ in this figure) in the half space of the normal (*N* in this figure) of the clip plane is finally preserved and used for splatting and shear warp.

- *Splatting:* voxels are splatted scanline by scanline in a sheet. For each scanline in the sheet, all segments out of the clip planes are removed (the clipping of scanline is described in “(C) Scanline Clipping” and illustrated in Figure 4). In the preserved segment, the color and opacity of each voxel are calculated. Then all voxels are projected onto the screen and the splats are added into a sheet buffer. After all scanlines within the sheet are processed, the sheet buffer is composited with an accumulated buffer by (5). The final projection image is obtained by traversing the volume from the nearest sheet to the farthest sheet.
- *Shear warp:* the volume is also traversed from the nearest sheet to the farthest sheet, and each sheet is traversed in scanline order as well. After each scanline in a sheet is clipped, its preserved segment is transformed to the shear space. Then each voxel in the segment is projected onto the plane of the intermediate image. The color and opacity of each voxel are distributed to a sheet buffer. After traversing all scanlines within the sheet, we composite the sheet buffer with the intermediate image according to (5). Once the intermediate image is formed after processing all sheets, the final projection image is obtained by projecting the intermediate image onto the screen.
- *Scanline clipping:* in *splatting* and *shear warp*, each scanline is clipped in the coordinate system G. Figure 4 illustrates the clipping of a scanline. Assume that (1) the origin and normal of the clip plane in the grid space are *O* and *N*, respectively; *F* and *L* are, respectively, the first and last voxels in the scanline; the scanline intersects against the clip plane at *K*; *V*_*S*_ and *V*_*E*_ are, respectively, the first and last voxels of the finally preserved segment. Then the scanline is clipped as follows.
  1. Let *V*_*S*_ = *F* and *V*_*E*_ = *L*.
  2. Calculate the dot product of $\vec{OF}$ and *N* by *D*_*F*_ = $\vec{OF}\cdot N$ and the dot product of $\vec{OL}$ and *N* by *D*_*L*_ = $\vec{OL}\cdot N$.
  3. If *D*_*F*_ > 0 and *D*_*L*_ < 0, let *V*_*E*_ = *K*. If *D*_*F*_ < 0 and *D*_*L*_ > 0, let *V*_*S*_ = *K*.
  4. If *D*_*F*_ > 0 and *D*_*L*_ > 0, the entire scanline is preserved and keep *V*_*S*_ = *F* and *V*_*E*_ = *L*.
  5. Else the entire scanline is removed and let *V*_*S*_ = 0 and *V*_*E*_ = 0.

#### 2.3.2. Rendering the Projection Image and 3D Plane Widgets

**Figure 5:**
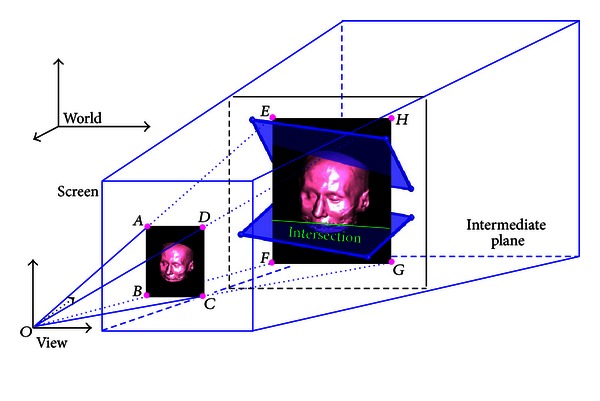
The rendering of the projection image and 3D plane widgets. The projection image is generated by projecting the clipped volume onto the screen using volume rendering. It is worth noting that the center of the volume is always located in the intermediate plane. The projection image is textured and mapped onto the intermediate plane, reconstructing the depth information of the projection image. This depth information is the basis to correctly display the combination of the projection image and 3D plane widgets.

Assume that (1)  *A*, *B*, *C*, and *D* (see Figure 5) are the four vertices of the projection image obtained in Section 2.3.1;(2)  *O* is the origin of *V*; (3)  *OA*, *OB*, *OC*, and *OD* intersect against the intermediate plane of the view space at *E*, *F*, *G*, and *H*, respectively. The projection image is then textured and mapped onto the rectangle *EFGH*. Finally, all widgets are rendered at exact positions. As shown in Figure 5, there may be an intersection error between the mapped texture of the projection image and each clip plane. By setting the clip planes to be completely transparent, we can eliminate the intersection errors. However, sometimes we also want to display the intersection images between the volume and clip planes, which can be achieved by the following steps.

1. After creating the texture *T*_*P*_ from the projection image, compute the intersection images between the volume and clip planes, and creating textures from the intersection images.
2. Map *T*_*P*_ onto the rectangle *EFGH* and render the 3D plane widgets with completely transparent clip planes.
3. For each 3D plane widget, map the texture of the intersection image onto the clip plane if the intersection image faces the view direction.

## 3. Experimental Results

**Figure 6:**
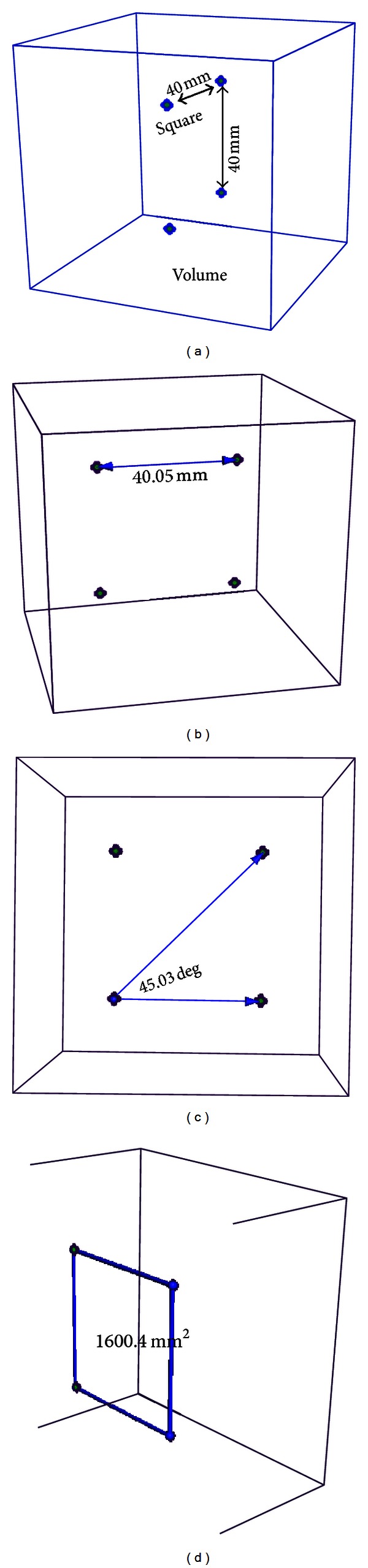
Validation of the accuracy of interactive 3D measurement. (a) The simulated volume with 4 highlighted voxels. (b) Distance measurement. (c) Angle measurement. (d) Area measurement.

A group of experiments are performed on a Windows PC (with an Intel Core2 1.86 GHz processor and 1 GB physical memory) to demonstrate the validity of our interactive 3D measurement framework. Firstly, a simulated volume with four highlighted voxels is tested for the validation of the accuracy of the interactive 3D measurement. Secondly, realistic CT and MR images are tested in the experiments. The simulated volume is shown in Figure 6(a). The simulation is described as below: (1) the volume size and voxel size are 81 × 81 × 81 and 1 × 1 × 1 mm^3^, respectively; (2) the values of the background, edges, and 4 highlighted voxels of the volume are 10, 50, and 255, respectively; (3) the 4 highlighted voxels make a square whose edge length is 40 mm. The realistic CT image is from the School of Psychology at the University of Nottingham, and the MR image is from Beijing Shougang Hospital. The size and spacing of the CT image are 208 × 256 × 225 and 1 × 1 × 1 (mm^3^), respectively. The size and spacing of the realistic MR image are 256 × 256 × 124 and 0.86 × 0.86 × 1.7 (mm^3^), respectively.

To validate the accuracy of the interactive 3D measurement, we interactively explore the simulated volume and measure the length of the edge, the half angle between the neighboring edges, and the area of the square by using the 3D line widget, 3D angle widget, and 3D plane widget, respectively. The measured length (40.05 mm), angle (45.03 deg), and area (1600.4 mm^2^) are very close to the simulated ones (40 mm, 45 deg, and 1600 mm^2^, resp.). The measurement errors are within 1 mm, 1 deg, and 1 mm^2^. It is worth noting that more accurate measurement results could be reached by interactively manipulating the 3D widgets more carefully. The experiments indicate that the interactive 3D measurement technology can achieve sufficient accuracy.

**Figure 7:**
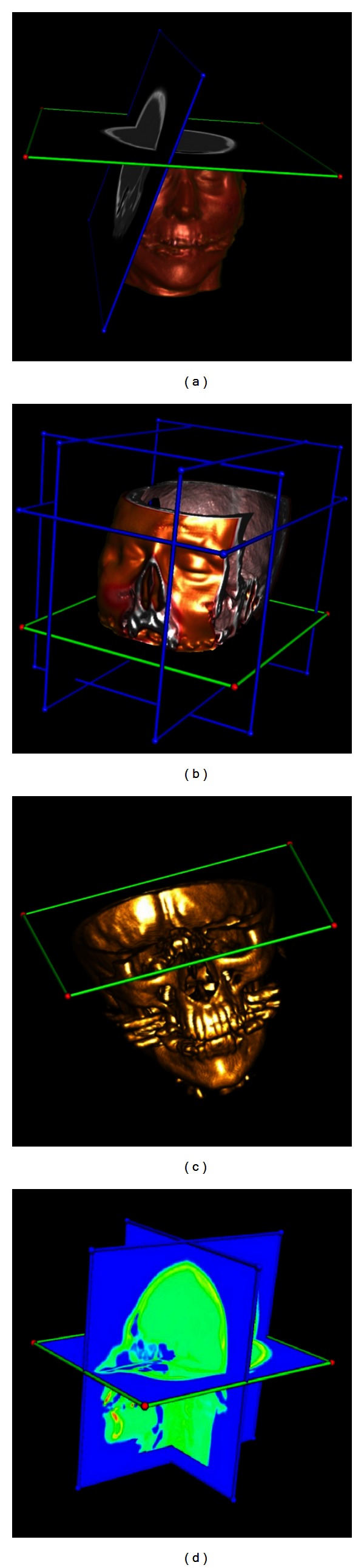
Interactive volume clipping. (a) Ray casting with reslicing. (b) Ray casting with cubic clipping. (c) Splatting without reslicing. (d) Shear warp with pseudocolor.

The CT image is interactively explored using 3D plane widgets.  Figure 7 shows representative volume clipping results based on *ray casting*, *splatting*, and *shear warp* renderers. In addition, the rendering efficiency is measured using the following parameters.

1. The screen size is 948 × 618. The volume model is rotated to a certain orientation to form a 290 × 386 projection image.
2. The volume is rendered without shading. Each intensity is mapped to (*r*, *g*, *b*, and *a*). We map 0 to (0.0, 0.0, 0.0, 0.0) and 255 to (0.8, 0.8, 0.2, 0.5). Other intensities are mapped by linear interpolation.
3. The half brain is clipped by a 3D plane widget and reslicing is enabled. Rough rendering is performed while the volume or widget is being manipulated, and refined rendering is performed while the manipulation is stopped.

The average frame rates of scene rendering (i.e., rendering of all models) under different circumstances are shown in Table 1.

**Figure 8:**
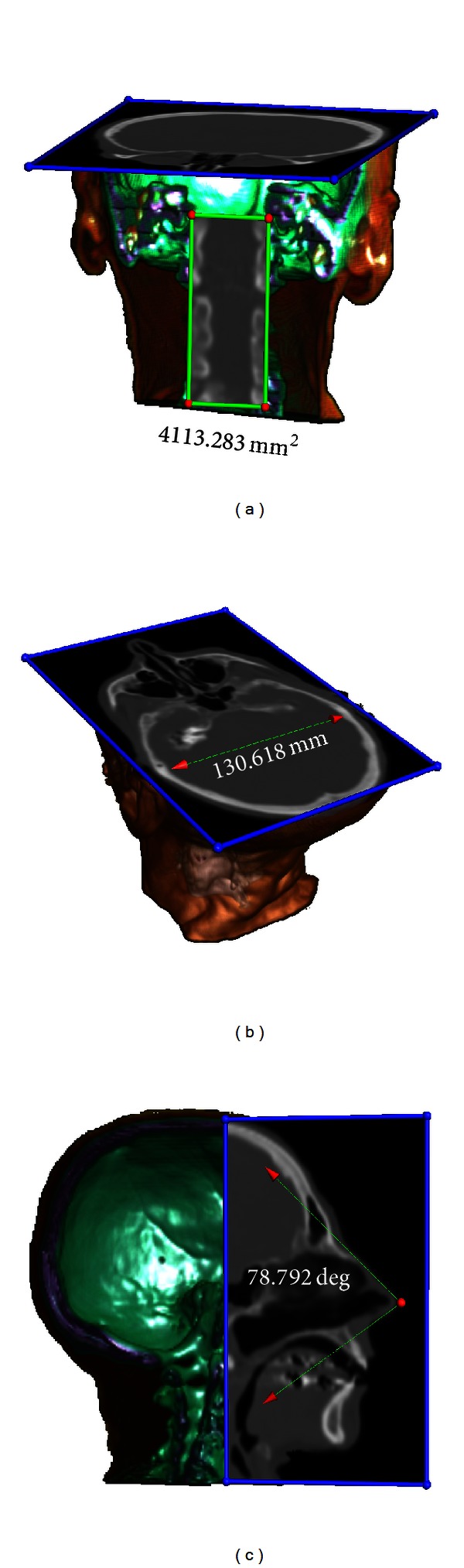
Interactive 3D measurement of the CT image. (a) Area measurement based on splatting. (b) Distance measurement based on ray casting. (c) Angle measurement based on shear warp.

**Figure 9:**
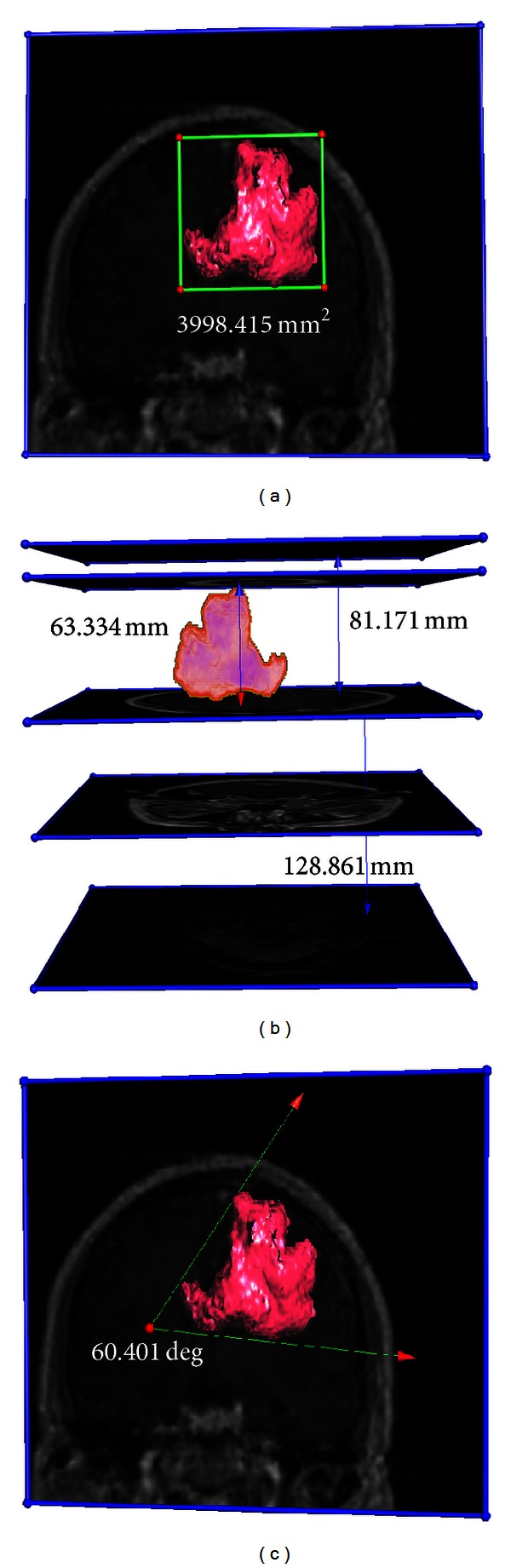
Interactive 3D measurement of the tumor in the brain MR image. First, the tumor is segmented from the MR image using a region growing method automatically. Then, the tumor model and the original MR image are put in the same rendering environment for interactive 3D measurement. (a) Area measurement (surface model of the tumor is used and rendered by surface rendering). (b) Distance measurement (volume model of the tumor is used and rendered by ray casting). (c) Angle measurement (surface model of the tumor is used and rendered by surface rendering).

The CT and MR images are interactively clipped and measured.  Figure 8 shows the interactive measurement results for the CT image.  Figure 9 shows the instances of measuring the position and size of the tumor in the brain MR image. 

## 4. Conclusion and Future Work

In this paper, an interactive 3D measurement framework based on volume rendering is implemented for quantitative analysis of 3D medical images. 3D plane widgets are manipulated to interactively clip the volume and expose interesting objects. The 3D plane widgets are also manipulated to measure the areas of interesting objects. Further, 3D line widgets and 3D angle widgets are manipulated to measure the distances and angles of interesting objects, respectively. The volume and 3D widgets can be manipulated interactively and intuitively as if they were manipulated by the operator in a real 3D space. With level-of-detail rendering, we can achieve satisfactory frame rates during manipulation and obtain rendering images of high quality after manipulation.

Various volume rendering algorithms (including ray-casting, splatting, and shear warp) are supported in our framework. Compared with these algorithms, the texture-based volume rendering algorithm is faster. However, for the texture-based algorithm, the rendering quality and the size of the rendered volume are restricted by the texture of the graphics hardware. Therefore, our framework is a necessary complement to the interactive 3D measurement using texture-based volume rendering [10, 12]. In addition, it is worth noting that the performance of our framework can be improved largely if the ray-casting, splatting, and shear-warp algorithms are implemented using the GPU.

Currently only 3D plane widgets are used for volume clipping. Although they can be combined to construct some clipping widgets (e.g., cube widgets), more useful clipping widgets are required to expose complex objects of interest. In the future, we will design more types of clipping widgets (such as sphere and cylinder widgets). Furthermore, we will design more practical 3D measurement widgets in addition to the 3D line and angle widgets. Our interactive 3D measurement framework has been integrated in the Medical Imaging ToolKit (MITK) [14], which can be downloaded freely from http://www.mitk.net.

## Figures and Tables

**Table 1:** Rendering efficiency of the volume clipping. It can be seen that interactive rendering rate can be achieved. It's worth noting that all the volume renderers in this paper are software-based. The rendering speed can be further accelerated if the volume renderers are implemented using the GPU.

Frames/second	Rough rendering during manipulation	Refined rendering
Ray casting	28.3	1.2
Splatting	9.1	0.5
Shear warp	37.4	2.6
